# The toxicities of A30P and A53T α-synuclein fibrils can be uniquely altered by the length and saturation of fatty acids in phosphatidylserine

**DOI:** 10.1016/j.jbc.2023.105383

**Published:** 2023-10-26

**Authors:** Abid Ali, Kiryl Zhaliazka, Tianyi Dou, Aidan P. Holman, Dmitry Kurouski

**Affiliations:** 1 Department of Biochemistry and Biophysics, Texas A&M University, College Station, Texas, USA; 2 Department of Entomology, Texas A&M University, College Station, Texas, USA; 3 Department of Biomedical Engineering, Texas A&M University, College Station, Texas, USA

**Keywords:** α-synuclein, phosphatidylserine, oligomers, fibrils, toxicity, AFM-IR

## Abstract

Progressive degeneration of dopaminergic neurons in the midbrain, hypothalamus, and thalamus is a hallmark of Parkinson’s disease (PD). Neuronal death is linked to the abrupt aggregation of α-synuclein (α-syn), a small protein that regulates vesicle trafficking in synaptic clefts. Studies of families with a history of PD revealed several mutations in α-syn including A30P and A53T that are linked to the early onset of this pathology. Numerous pieces of evidence indicate that lipids can alter the rate of protein aggregation, as well as modify the secondary structure and toxicity of amyloid oligomers and fibrils. However, the role of lipids in the stability of α-syn mutants remains unclear. In this study, we investigate the effect of phosphatidylserine (PS), an anionic lipid that plays an important role in the recognition of apoptotic cells by macrophages, in the stability of WT, A30P, and A53T α-syn. We found PS with different lengths and saturation of fatty acids accelerated the rate of WT and A30P aggregation. At the same time, the opposite effect was observed for most PS on A53T. We also found that PS with different lengths and saturation of fatty acids change the secondary structure and toxicities of WT, A30P, and A53T fibrils. These results indicate that lipids can play an important role in the onset and spread of familial PD.

Abrupt aggregation of α-synuclein (α-syn), a small membrane protein that regulates vesicle trafficking in synaptic clefts, is the expected underlying molecular cause of Parkinson's disease (PD) (1, 2, 3). Primarily, because α-syn oligomers and fibrils are found in Lewy bodies, extracellular formations are observed in the midbrain, hypothalamus, and thalamus of individuals diagnosed with PD (4, 5, 6, 7, 8). DNA sequencing of individuals with familiar cases of PD revealed several mutations, including A30P and A53T that are linked to the early onset of PD, Figure 1 (9, 10, 11, 12). Recently reported results by the Galvagnion group demonstrated that 1,2-dimyristoyl-*sn*-glycero-3-phospho-Lserine (DMPS) could uniquely alter the aggregation rate of WT α-syn, as well as its A30P and A53T mutants (13). Specifically, it was shown that DMPS could accelerate the aggregation rate of WT, A30P, and A53T. A growing body of evidence indicates that the effect of the phospholipid on the rate of protein aggregation can be uniquely attenuated by the length and saturation of its fatty acids (FAs) (14, 15, 16, 17, 18, 19, 20). Matveyenka and co-workers found that cardiolipin with unsaturated FAs caused a much stronger enhancement of insulin aggregation than cardiolipin with fully saturated FAs (14, 15, 17). It was also shown that the saturation and length of FAs in both phosphatidylserine (PS) and phosphatidic acid uniquely altered the aggregation rate of insulin (14, 15, 17). However, it remains unclear whether the length and saturation of FAs in PS could alter the aggregation rate of WT α-syn, as well as its A30P and A53T mutants.

**Figure 1:**
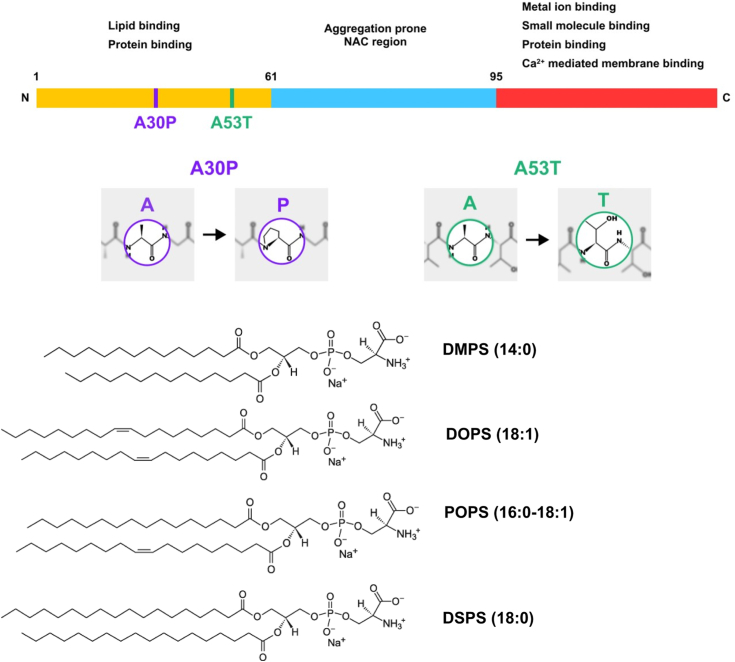
**A schematic diagram of α-syn sequence with indicated A30P and A53T mutations (*top*) and molecular structures of DMPS, DOPS, POPS, and DSPS (*bottom*).** α-syn, α-synuclein; DMPS, 1,2-dimyristoyl-*sn*-glycero-3-phospho-Lserine; DOPS, 1,2-dioleoyl-*sn*-glycero-3-phospho-L-serine; DSPS, 1,2-distearyl-*sn*-glycero-3-phospho-L-serine; POPS, 1-palmitoyl-2-oleoyl-*sn*-glycero-3-phospho-L-serine.

To end this, we investigated the extent to which equimolar concentrations of DMPS, 1,2-dioleoyl-*sn*-glycero-3-phospho-L-serine (DOPS), 1-palmitoyl-2-oleoyl-*sn*-glycero-3-phospho-L-serine (POPS), and 1,2-distearyl-*sn*-glycero-3-phospho-L-serine (DSPS), could change the aggregation rate of WT α-syn, as well as its A30P and A53T α-syn mutants, Figure 1. We also utilize a set of biophysical methods to determine the morphology and secondary structure of WT, A30P, and A53T α-syn fibrils formed in the lipid-free environment and the presence of DMPS, DSPS, DOPS, and POPS. Finally, a lactate dehydrogenase (LDH)-based cell toxicity assay was employed to elucidate the extent to which phospholipids altered the toxicity of WT, A30P, and A53T α-syn fibrils.

This study is significant because PS is an anionic lipid that is localized in the inner part of the plasma membrane *via* ATP-dependent flippase transport (21, 22). An increase in the concentration of PS on the outer surface of the plasma membrane is recognized by macrophages which can trigger apoptosis (17, 23). One can expect that the presence of PS in the outer membrane can drastically alter the stability of misfolded proteins which can stimulate the aggregation of WT, A30P, and A53T α-syn and, consequently, trigger the onset of PD (17, 20, 24, 25).

## Results

### Elucidation of the effect of the length and saturation of FAs in PS on the aggregation rate of WT α-syn, A30P, and A53T mutants

We used thioflavin T (ThT) assay to reveal the extent to which length and saturation of FAs in PS alter the rate of aggregation of WT α-syn, A30P, and A53T mutants. In the absence of protein aggregates, ThT is not fluorescent. However, in the presence of protein oligomers and fibrils, ThT binds to their surfaces, which results in a drastic increase in ThT fluorescence. Therefore, a change in ThT fluorescence can be used to measure the rate of protein aggregation.

**Figure 2:**
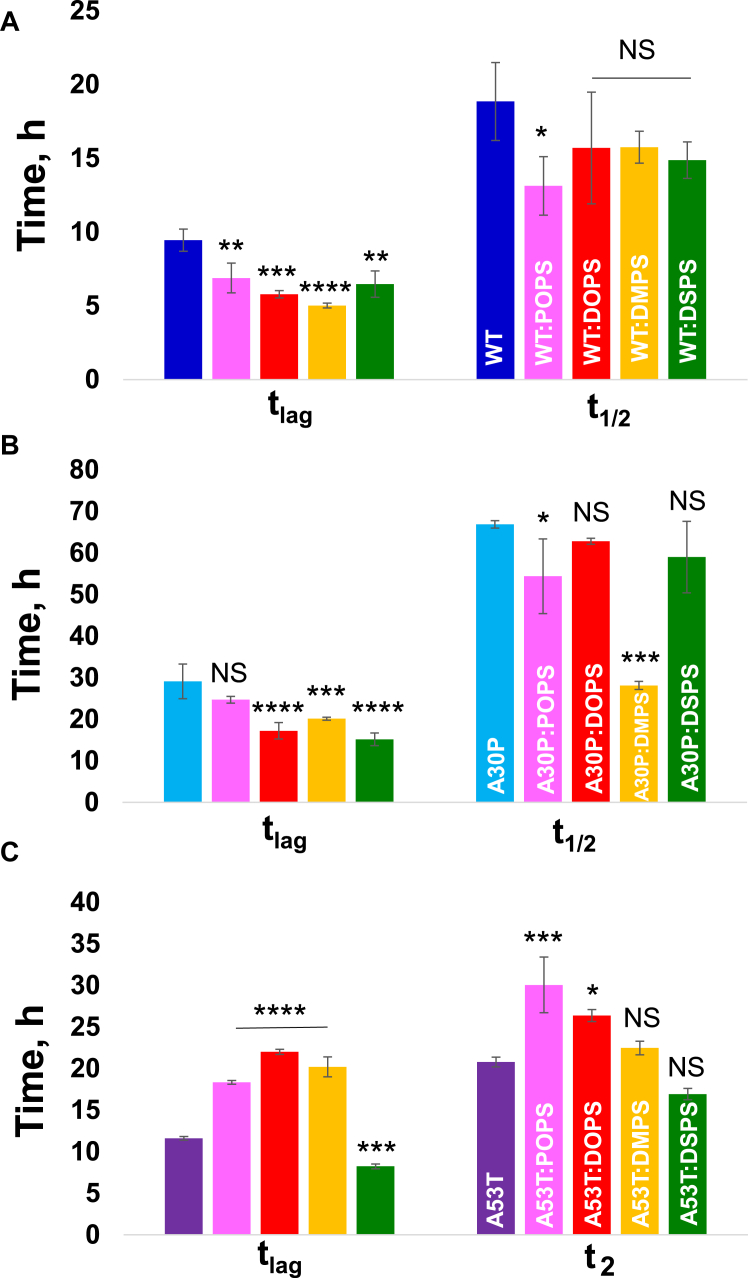
**Saturation and lengths of FAs in phosphatidylserine uniquely alter the rate of WT, A30P, and A53T α-syn aggregation.** ThT aggregation kinetics of (*A*) WT, (*B*) A30P, and (*C*) A53T aggregation in the lipid-free environment (*blue*, WT; *light blue*, A30P; and *purple*, A53T) and in the presence of POPS (*pink*), DOPS (*red*), DMPS (*yellow*) and DPSP (*green*) at 1:1 M ratio. Bar graphs that summarize the corresponding t_lag_ and t_1/2_ indicate aggregation time when the intensity of ThT fluorescence reached 10% (t_lag_) and 50% (t_1/2_) of the maximal ThT intensity observed for each sample. Each kinetic curve is the average of four independent measurements. One-way ANOVA with Tukey’s honestly significant difference post hoc was performed to reveal statistical significance between all groups. NS is a nonsignificant difference; ∗*p* ≤ 0.05, ∗∗*p* ≤ 0.01, ∗∗∗*p* ≤ 0.001, and ∗∗∗∗*p* ≤ 0.0001. α-syn, α-synuclein; DMPS, 1,2-dimyristoyl-*sn*-glycero-3-phospho-Lserine; DOPS, 1,2-dioleoyl-*sn*-glycero-3-phospho-L-serine; DSPS, 1,2-distearyl-*sn*-glycero-3-phospho-L-serine; FA, fatty acid; POPS, 1-palmitoyl-2-oleoyl-*sn*-glycero-3-phospho-L-serine; ThT, thioflavin T.

In the lipid-free environment, WT α-syn exhibited a well-defined lag phase (t_lag_ = 9.0 ± 0.5 h) that was followed up by a rapid increase in ThT fluorescence. We found that both POPS and DOPS accelerated the rate of WT α-syn aggregation, t_lag_ = 6.8 ± 1.1 h, and t_lag_ = 5.2 ± 0.6 h, respectively, Figure 2 and Figs. S1 and S2. We found that both saturated PS, DMPS, and DSPS also strongly accelerated the rate of WT aggregation (t_lag_ = 4.7 ± 0.2 h and t_lag_ = 6.2 ± 0.8 h, respectively). Thus, we can conclude that anionic PS strongly accelerated the rate of WT aggregation by facilitating the primary nucleation step. Our results also showed that both the length of FAs in PS, as well as their saturation, uniquely altered the rate of protein aggregation. Specifically, DMPS, the saturated PS with the shortest FA length, exerted the strongest acceleration effect on WT α-syn aggregation compared to all other PSs.

Our results also showed that PSs only altered the onset of protein aggregation (t_lag_), however, exerted very little, if any, effect on the rate of protein aggregation (t_1/2_), Figure 2 and Figs. S1 and S2. Specifically, we found that only POPS accelerated the growth of WT fibrils, whereas this effect was not observed for any other lipids. Thus, one can expect that PSs only interacted with monomeric WT, which assists monomeric protein to assemble and form nuclei and oligomers with the addition of nuclei. However, once the oligomers were formed, DOPS, DMPS, and DSPS did not alter the rate of their growth.

A similar effect of PSs was observed for A30P. Specifically, we found that DOPS, DMPS, and DSPS strongly shortened the lag phase of A30P aggregation. However, no effect on the lag phase of A30P oligomerization was observed for POPS. We also found that POPS, similar to WT, accelerated the rate of A30P aggregation, whereas DOPS and DSPS did not affect the rate of A30P fibril growth. Our results also showed that the presence of DMPS strongly accelerated the rate of A30P fibrillization. Thus, we can conclude that the length and saturation of FAs in PS uniquely alter both the lag phase and the rate of A30P aggregation. Our results also showed that the effect exerted by the same lipid was different for A30P and WT α-syn.

Interestingly, the same phospholipids exerted drastically different effects on the rate of A53T aggregation. We found that POPS, DOPS, and DMPS solutions delayed protein aggregation, whereas DSPS shortened the lag phase of A53T aggregation. We also found that although DMPS and DSPS did not alter the rate of protein aggregation, POPS and DOPS significantly decelerated the rate of A53T aggregation. Based on these results, we can conclude that changes in the length and saturation of FAs in PS uniquely alter the rate of WT, A30P, and A53T α-syn aggregation.

### Morphological characterization of WT, A30P, and A53T α-syn fibrils formed in the presence of POPS, DOPS, DMPS, and DSPS

**Figure 3:**
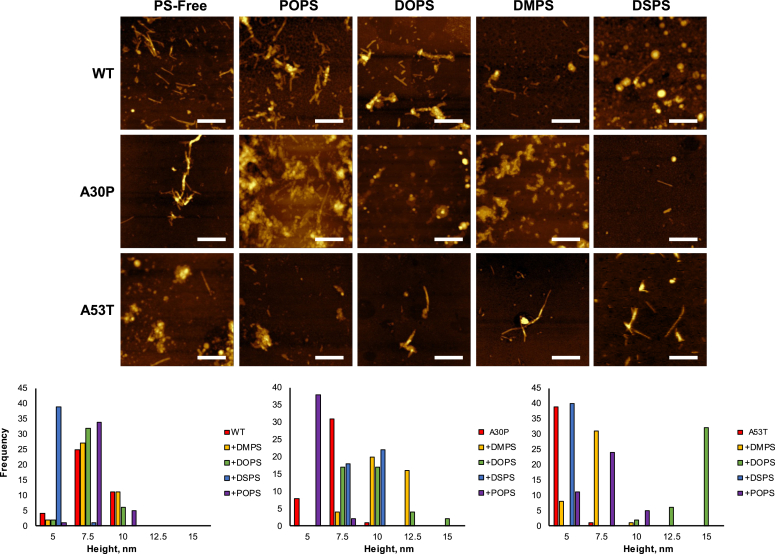
**AFM images (*top*) of WT, A30P, and A53T α-syn aggregates formed in the lipid-free environment (PS-free), as well as in the presence of POPS, DOPS, DMPS, and DSPS.***White scale bars* are 500 nm. Histograms (*bottom*) of height distribution of fibrils observed by AFM. α-syn, α-synuclein; AFM, atomic force microscopy; DMPS, 1,2-dimyristoyl-*sn*-glycero-3-phospho-Lserine; DOPS, 1,2-dioleoyl-*sn*-glycero-3-phospho-L-serine; DSPS, 1,2-distearyl-*sn*-glycero-3-phospho-L-serine; POPS, 1-palmitoyl-2-oleoyl-*sn*-glycero-3-phospho-L-serine; PS, phosphatidylserine.

Atomic force microscopy (AFM) imaging revealed that in the lipid-free environment, WT formed thin fibrils (5–11 nm) that stretched microns in length. We also found a small amount of protein oligomers remained in this sample after 160 h of protein aggregation. Morphologically similar fibrils were observed in A30P. These aggregates were 5 to 8 nm in height. At the same time, A53T fibrils were substantially thinner (3.2–4.8 nm), Figure 3. We also observed a greater number of oligomeric species in A53T. AFM imaging demonstrated that POPS, DOPS, DMSP, and DSPS had a minor effect on the morphology of WT α-syn fibrils. We found that the heights and overall shape of the fibrils remained unchanged except for WT:DMPS, which exhibited slightly thinner fibrils with heights ranging from 3.2 to 4.8 nm. We also found that the presence of POPS, DOPS, and DMPS resulted in a small increase in the thickness of A30P fibrils, with heights that slightly increased to 7 to 12 nm compared to A30P itself (3–8 nm), Figure 3. At the same time, we found that A30P formed slightly skinnier fibrils in the presence of DSPS (3.5–4.3 nm). We also found that the overall shape and morphology of A30P fibrils changed if those were formed in the presence of DMPS compared to A30P fibrils. Finally, we found that A30P formed very little if any fibrils in the presence of DOPS. The same conclusion can be made about A53T fibrils. We found that only in the presence of POPS, DOPS, and DSPS, the heights of these aggregates slightly increase. Based on these results, we can conclude that POPS, DOPS, DMPS, and DSPS caused only small changes to the height of protein fibrils formed in their presence, whereas the overall shape of these aggregates remains unchanged compared to the fibrils formed by WT, A30P, and A53T in the lipid-free environment. Thus, changes in the length and saturation of FAs in PSs did not significantly change the morphology of protein aggregates.

### Structural characterization of WT, A30P, and A53T fibrils formed in the presence of POPS, DOPS, DMPS, and DSPS

We utilized attenuated total reflectance (ATR)-FTIR and CD to examine the secondary structure of protein aggregates present in WT, A30P, and A53T themselves, as well as the fibrils formed by these proteins in the presence of POPS, DOPS, DMPS, and DSPS.

**Figure 4:**
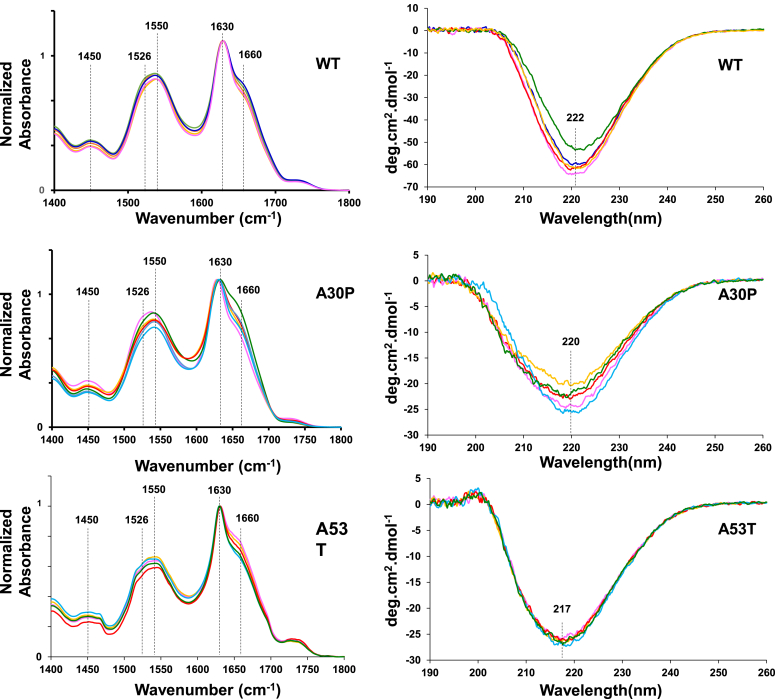
**ATR-FTIR (*left*) and CD (*right*) spectra of WT, A30P, and A53T α-syn aggregates formed in the lipid-free environment (PS-free), as well as in the presence of POPS, DOPS, DMPS, and DSPS.** α-syn, α-synuclein; ATR, attenuated total reflectance; DMPS, 1,2-dimyristoyl-*sn*-glycero-3-phospho-Lserine; DOPS, 1,2-dioleoyl-*sn*-glycero-3-phospho-L-serine; DSPS, 1,2-distearyl-*sn*-glycero-3-phospho-L-serine; POPS, 1-palmitoyl-2-oleoyl-*sn*-glycero-3-phospho-L-serine; PS, phosphatidylserine.

Our results demonstrated that all acquired ATR-FTIR spectra from WT, WT:POPS, WT:DOPS, WT:DMPS, and WT:DSPS exhibited C-H (1460 cm^−1^), amide II (1526–1550 cm^−1^), and amide I (1630–1660 cm^−1^) vibrations. In the acquired spectra, amide I was centered around 1630 cm^−1^, which indicates the predominance of parallel β-sheet in the secondary structure of analyzed samples, Figure 4 (14, 15, 17). We also found a shoulder around 1660 cm^−1^, which points to the presence of some unordered protein in WT, WT:POPS, WT:DOPS, WT:DMPS, and WT:DSPS. Similar IR spectra were acquired from A30P and A53T aggregates formed in the presence of POPS, DOPS, DMPS, and DSPS. These findings show that WT, A30P, and A53T fibrils formed in the presence of POPS, DOPS, DMPS, and DSPS were dominated by parallel β-sheet with some amount of unordered protein present in these samples. The same conclusions could be made based on the acquired CD spectra. Specifically, we found that all acquired spectra from WT, WT:DOPS, WT:POPS, WT:DMPS, and WT:DSPS had a trough at ∼222 nm, which indicates the predominance of β-sheet in the secondary structure of analyzed samples (14, 15, 17). All fibrillar species formed by A30P and A53T in the presence of PSs with different lengths and saturation of FAs exhibited similar CD spectra with a trough at 220 nm and 217 nm, respectively, Figure 4.

It should be noted that both ATR-FTIR and CD probe the bulk volume of analyzed samples, which in addition to amyloid fibrils and oligomers has a substantial amount of unaggregated protein. To overcome this limitation, we utilized nano-IR spectroscopy, also known as AFM-IR spectroscopy. In AFM-IR, a metalized scanning probe can be positioned directly at the object of interest. Next, the sample is illuminated by pulsed tunable IR light that causes thermal expansions in the sample. These thermal expansions are then recorded by the scanning probe and converted into IR spectra. A growing body of evidence indicates that AFM-IR could be used to unravel the secondary structure of individual proteins, protein oligomers, and fibrils, as well as epicuticular waxes, cells, and polymer films (26, 27, 28, 29, 30, 31, 32, 33, 34, 35, 36, 37, 38, 39, 40, 41).

**Figure 5:**
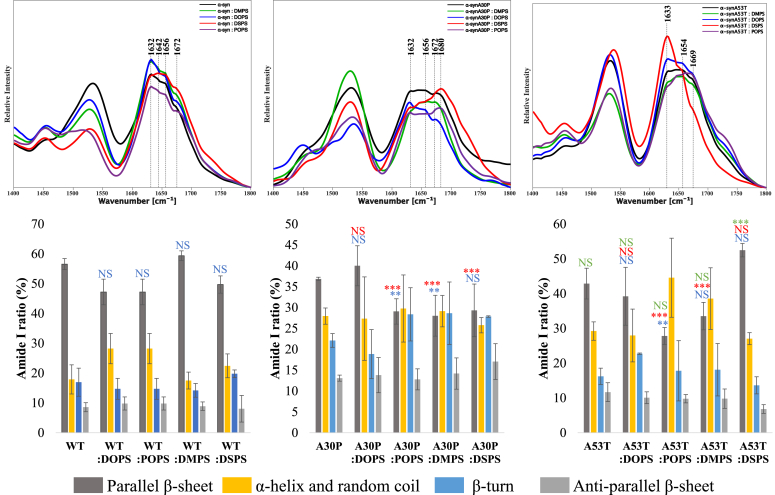
**AFM-IR spectra (*top*) of WT, A30P, and A53T α-syn aggregates formed in the lipid-free environment (PS-free), as well as in the presence of POPS, DOPS, DMPS, and DSPS with corresponding bar graphs (*bottom*) showing the amount of parallel β-sheet (*gray*), α-helix and random coil (*yellow*), β-turn (*blue*), and antiparallel β-sheet (*light gray*) in these aggregates.** One-way ANOVA with Tukey’s honestly significant difference post hoc was performed to reveal statistical significance between all groups. ∗*p* ≤ 0.05, ∗∗*p* ≤ 0.01, ∗∗∗*p* ≤ 0.001, and ∗∗∗∗*p* ≤ 0.0001. For the ∗*P*, comparing with lipid-free environment in *blue*; comparing with WT in *red*; comparing with A30P in *green*. α-syn, α-synuclein; AFM, atomic force microscopy; DMPS, 1,2-dimyristoyl-*sn*-glycero-3-phospho-Lserine; DOPS, 1,2-dioleoyl-*sn*-glycero-3-phospho-L-serine; DSPS, 1,2-distearyl-*sn*-glycero-3-phospho-L-serine; NS, nonsignificant difference; POPS, 1-palmitoyl-2-oleoyl-*sn*-glycero-3-phospho-L-serine; PS, phosphatidylserine.

AFM-IR analysis of WT fibrils showed that these protein aggregates had ∼57% and 10% of parallel and antiparallel β-sheet, respectively, Figure 5 and Fig. S3. We also found that WT fibrils possessed around 15% of α-helix, β-turn, and random coil in their secondary structure. Our results showed that the secondary structure of A53T fibrils was very similar if not identical to WT α-syn. These results are in good agreement with experimental findings that were previously reported by Ruggeri and co-workers. However, we found that A30P fibrils had a significantly lower amount of parallel β-sheet than both WT and A53T, simultaneously possessing higher amount of antiparallel β-sheet, α-helix, β-turn, and random coil in their secondary structure.

AFM-IR revealed that the length and saturation of FAs in PSs uniquely altered the secondary structure of protein aggregates that were grown in their presence. Specifically, we found that A30P:POPS fibrils had a significantly lower amount of parallel β-sheet than WT fibrils. A30P:POPS fibrils possessed a greater amount of antiparallel β-sheet, α-helix, β-turn, and random coil in their secondary structure than WT α-syn fibrils. We also found high structural similarity between A30P:POPS and A53T:POPS fibrils. However, sharing the similar content of parallel β-sheet and α-helix and random coil, A53T:POPS fibrils possess lower amount of antiparallel β-sheet and a higher amount of β-turns than A30P:POPS fibrils. Our fibrils also showed that the secondary structure of WT:DOPS, A30P:DOPS, and A53T:DOPS were identical. At the same time, we found that PSs with saturated FAs drastically changed the secondary structure of A30P and A53T fibrils compared to the structure of WT fibrils that were formed in their presence. Specifically, A30P:DMPS fibrils possessed a significantly higher amount of parallel β-sheet than both WT:DMPS and A53T:DMPS. At the same time, these aggregates had higher amounts of α-helix, β-turns, and random coil than WT:DMPS fibrils. Finally, we found that A30P:DSPS had significantly lower, whereas A53T:DSPS had significantly higher amount of parallel β-sheet than WT fibrils. These fibrils had higher amounts of α-helix, β-turn, and random coil, as well as antiparallel β-sheet than both WT:DSPS fibrils. We also found a lower amount of β-turn and anti-parallel β-sheet in A53T:DSPS than A30P:DSPS. These results demonstrated that the length and saturation of FAs in PS uniquely altered the secondary structure of WT, A30P, and A53T fibrils.

### Toxicity of WT, A30P, and A53T fibrils formed in the presence of POPS, DOPS, DMPS, and DSPS

**Figure 6:**
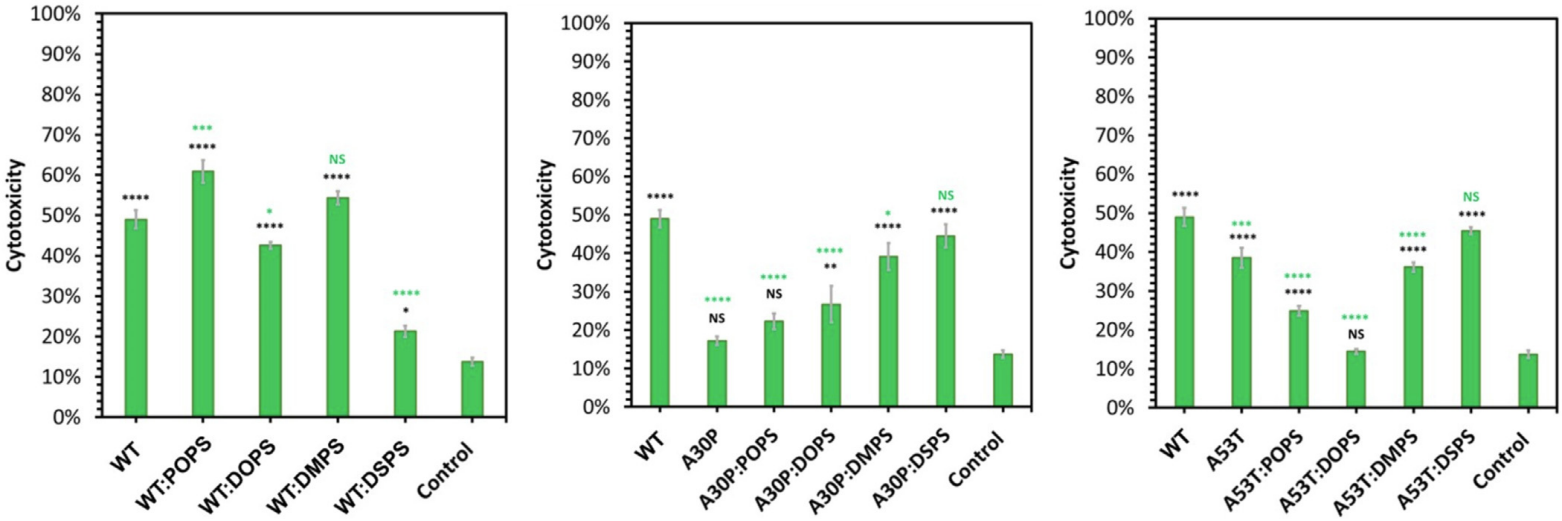
**Histograms of LDH assays of WT, A30P, and A53T α-syn aggregates formed in the lipid-free environment (PS-free), as well as in the presence of POPS, DOPS, DMPS, and DSPS.** For each of the presented results, three independent measurements were made. One way ANOVA with Tukey’s honestly significant difference post hoc was performed to reveal statistical significance between all groups. ∗*p* ≤ 0.05, ∗∗*p* ≤ 0.01, ∗∗∗*p* ≤ 0.001, and ∗∗∗∗*p* ≤ 0.0001. α-syn, α-synuclein; DMPS, 1,2-dimyristoyl-*sn*-glycero-3-phospho-Lserine; DOPS, 1,2-dioleoyl-*sn*-glycero-3-phospho-L-serine; DSPS, 1,2-distearyl-*sn*-glycero-3-phospho-L-serine; LDH, lactate dehydrogenase; NS, nonsignificant difference; POPS, 1-palmitoyl-2-oleoyl-*sn*-glycero-3-phospho-L-serine; PS, phosphatidylserine.

Next, we investigated whether the length and saturation of FAs in PS could alter the toxicity of WT, A30P and A53T α-syn fibrils. For this, we performed an LDH assay on N27 rat dopaminergic cells exposed to these fibrillar species for 24 h. Our results showed that WT fibrils exerted significantly high (50%) cell toxicity, Figure 6 and Fig. S4. We also found that WT fibrils formed in the presence of DOPS and DSPS exerted significantly lower cell toxicity than the WT fibrils formed in the lipid-free environment. At the same time, fibrils formed in the presence of POPS by WT α-syn were found to be significantly higher than WT, WT:DOPS, and WT:DSPS fibrils. These results demonstrated that the length and saturation of FAs in PS uniquely altered the toxicity of WT α-syn fibrils. Similar conclusions could be made about the role of the length and saturation of FAs in PS for A30P. Specifically, we found that A30P:DOPS, A30P:DMPS, and A30P:DSPS fibrils were far more toxic than A30P fibrils. Thus, our results showed that the toxicity of A30P fibrils could be uniquely altered by the length and saturation of FAs in PS. It should be noted that our results revealed that A30P fibrils were significantly less toxic than WT fibrils to N27 rat dopaminergic cells. The same conclusion could be made about A53T fibrils. We also found that the toxicity of A53T fibrils could be uniquely altered by the length and saturation of FAs in PS. Specifically, we found A53T:POPS and A53T:DOPS were far less toxic to N27 rat dopaminergic cells compared to A53T fibrils formed in the lipid-free environment, Figure 6 and Fig. S4. We also found that A53T:DSPS fibrils were more toxic than all other protein aggregates formed by A53T.

## Discussion

The onset and spread of PD are linked to the aggregation of α-syn in the midbrain, hypothalamus, and thalamus. α-Syn is a small cytosolic protein that is mainly located in synaptic terminals (22). Although the physiologic function of α-Syn remains largely unknown (42, 43, 44), this protein plays an important role in synaptic plasticity, inflammatory response, and control of neurotransmitter release in synaptic clefts (45, 46). In solution, α-syn is an intrinsically disordered protein that adopts an α-helical structure in the presence of lipids (47, 48). α-Syn can interact with lipid bilayers, following two-dimensional surface restriction principle (49). NMR and fluorescence spectroscopy revealed that protein–lipid binding was determined by electrostatic interactions that were taken place between lysine and glutamic acid residues on the N terminus (1–60 aa) of α-syn and lipid headgroups (50). α-Syn–lipid interactions were also enhanced by hydrophobic interactions that took place between FAs of lipids and the central domain (61–95 aa) of α-Syn (51). These complexes alter the catalytic activity of cytoplasmic lipid enzymes and lysosomal lipases, which results in PD-specific alterations of lipids in both the brain and plasma (52). We infer that such two-dimensional α-syn–large unilamellar vesicle (LUV) interactions determined the observed changes in the rate of protein aggregation (49).

Our results showed that the length and saturation of FAs in PS uniquely altered the lag-phase and the aggregation rate of WT, A30P, and A53T α-syn, Table 1. Although all phospholipids shortened the lag-phase of WT aggregation, DMPS had the strongest, whereas POPS and DSPS had the weakest effect on the lag-phase of WT α-syn. It should be noted that only POPS accelerated the rate of WT aggregation, whereas this effect was not observed for POPS, DMPS, and DSPS. Similar effects these phospholipids exerted on A30P. However, we found that POPS did not significantly change the length of the lag-phase of A30P aggregation. However, POPS and DMPS drastically increased the rate of A30P aggregation compared to DOPS and DSPS. The opposite effect of these lipids was observed for A53T, Table 1. Specifically, we found that DOPS, POPS, and DMPS increased, whereas DSPS shortened the length of the lag-phase of A53T. We also found that POPS and DOPS decelerated the rate of A53T aggregation, whereas the same effect was not observed for DMPS and DSPS. Thus, we can conclude that the length and saturation of FAs in PS uniquely altered the aggregation rate of WT, A30P, and A53T α-syn. These results are in good agreement with the experimental findings that were previously reported by Matveyenka and co-workers for insulin (17). However, unlike in the case of insulin aggregates, we observed very little if any effect of differences in the length and saturation of FAs in PS on the morphology of α-syn fibrils, Table 1. At the same time, utilization of AFM-IR allowed for revealing small differences in the secondary structure of WT, A30P, and A53T α-syn fibrils formed in the lipid-free environment and in the presence of PSs with the different length and saturation of FAs, Table 1. We infer that these differences in the secondary structure of amyloid fibrils caused observed differences in the toxicity that these protein aggregates. Our results also demonstrated that A30P mutant exhibited much greater lag-phase than WT, which is consistent with the experimental results reported by Flagmeier and co-workers (13) and Lemkau and co-workers (53). However, we did not observe significant differences between the lag-phase aggregation of WT and A53T that were observed by Conway and co-workers in the presence of fibril seeds (54).

**Table 1:** Summary of the observed changes in t_lag_, t_1/2_, size of WT α-syn, A30P, and A53T fibrils, the amount of parallel β-sheet and toxicity of these protein aggregates according to LDH assay

	WT				A30P				A53T			
	POPS	DOPS	DMPS	DSPS	POPS	DOPS	DMPS	DSPS	POPS	DOPS	DMPS	DSPS
t_lag_	↓	↓	↓	↓	x	↓	↓	↓	↑	↑	↑	↑
t_1/2_	↓	x	x	x	↓	x	↓	x	↑	↑	x	x
Size												
Parallel β-sheet	x	x	x	x	x	↓	↓	↓	x	x	x	↑
Toxicity	↑	↓	x	↓	x	↑	↑	↑	↓	↓	↓	x

α-syn, α-synuclein; DMPS, 1,2-dimyristoyl-*sn*-glycero-3-phospho-Lserine; DOPS, 1,2-dioleoyl-*sn*-glycero-3-phospho-L-serine; DSPS, 1,2-distearyl-*sn*-glycero-3-phospho-L-serine; LDH, lactate dehydrogenase; POPS, 1-palmitoyl-2-oleoyl-*sn*-glycero-3-phospho-L-serine.

Previously reported results by Matveyenka and co-workers demonstrated that both oligomers and fibrils could be endocytosed by cells (18). Alternatively, protein aggregates may directly permeabilize lipid bilayers (55). In the former case, oligomers and fibrils damage endosomes and leak out to the cytosol, where they impair the physiological function of the endoplasmic reticulum and mitochondria. In the latter case, protein aggregates generate ROS, which ultimately leads to cell death. Matveyenka and co-workers found that protein aggregates formed in the presence of lipids and lipid-free environment exert drastically different cell toxicity to N27 cells (18). It was also reported that insulin and lysozyme aggregates formed in the presence of LUVs possessed lipids in their structure. One can expect that the presence of lipids on the surface of oligomers and fibrils drastically changed their interactions with the cell membranes, which ultimately lead to the observed differences in the oligomers’ and fibrils’ toxicity. At the same time, Zhaliazka and co-workers recently demonstrated a direct relationship between the amount of β-sheet and the toxicity of amyloid β_1-42_ aggregates (19). Thus, one can expect that differences in the secondary structure of amyloid aggregates determine their toxicity. Our current results show that likely both factors, the presence of lipids and protein secondary structure determine the toxicity of lysozyme aggregates formed in the presence of DMPS, DOPS, POPS, and DSPS, Table 1.

The observed differences in the aggregation rate of A30P and A53T point to the importance of electrostatic interactions that take place between charged amino acids in the N terminus (aa 1–60) of the protein and polar lipid heads (50). Our results suggest that point mutations alter these interactions, which result in the observed differences in the effect of PS on the rate of protein aggregation. Although the reported results by Flagmeier and co-workers (13) suggested that binding affinity of PS to folded WT, A30P and A53T is largely similar, protein misfolding could drastically alter such interactions. Thus, misfolded or partially unfolded WT, A30P and A53T may have drastically different surface electrostatics which would result in the observed difference in the rates of WT, A30P and A53T aggregation in the presence of the same phospholipid.

One can also expect that length and saturation of FAs in PS alter phospholipid ordering in LUVs. Lipid bilayers with unsaturated FAs typically have significantly lower order and much higher fluidity than lipid membranes with fully saturated FAs. Therefore, plasma membranes of mammalian cells possess very little amounts of DMPS and DSPS (21). Furthermore, previously reported simulation results by Dou and co-workers demonstrated that lipids adopted drastically different confirmations in lipid bilayers compared to those observed for free lipids (56). Thus, differences in the PS packing in the LUVs, which is determined by the length and saturation of FAs, can explain the discussed above differences in the rate of WT, A30P and A53T aggregation.

## Experimental procedures

### Materials

DMPS, DOPS, POPS, and DSPS were purchased from Avanti (Alabaster), IPTG was purchased from SIGMA

### Cloning and site-directed mutagenesis of the α-syn, A30P, and A53T

Plasmid pET-21a aSYN gene fragments were used as a mutagenesis template. All primers were designed to introduce the site-directed mutation at A30P and A53T specific position for A30P-F AGCACCAGGAAAGACAAAAGAGG G and A30P-R-TTCCTGGTGCTTCTGCCACACCC. In this study, we utilized plasmid pET-21a alpha-synuclein (aSYN) clone (pET 21a Asyn) as a template for mutagenesis. Two site-directed mutations, A30P and A53T, were introduced using carefully designed primers: A30P-F (AGCACCAGGAAAGACAAAAGAGG) and A30P-R (TTCCTGGTGCTTCTGCCACACCC) for A30P, and A53T-F (TGTGACAACAGTGGCTGA GAAG) and A53T-R (GTTGTCACACCATGCACCACTC) for A53T. These primers targeted specific positions in the aSYN gene to induce the desired mutations. The plasmids generated with these mutations can be employed for subsequent experiments, such as protein expression and purification. The 50 ul PCR reaction was carried out with 50 ng templates, 2 mM primer pair, 200 mM dNTPs, and 2 U of DNA fusion polymerase. The PCR amplification products were evaluated by 1% agarose gel electrophoresis. The PCRs were purified by Pure Link PCR Purification Kit (Thermo Fisher Scientific Inc) and further treated with restriction enzyme DpnI (NEB). An aliquot of 5 μl above PCR product was transformed into DH5α competent *Escherichia coli* cells and inoculated on Luria–Bertani plate containing 100 mg/ml ampicillin. A total of ten colonies were selected and their plasmids were isolated by mini prep (Thermo Fisher Scientific Inc). The positive mutants were selected by respective restriction enzyme (NdeI and XhoI) digestion. Mutants’ plasmid was sequenced by Eurofin to final confirmation on the mutations.

### Protein expression and purification α-syn, A30P and A53T

pET21a-α-syn as well as A30P and A53T was overexpressed in *E. coli BL21 (DE3)* Rosetta strain using LB broth media according to the protocol described by Volles and Lansbury (57, 58). Two liters of the bacterial culture (1 mM IPTG-induced) was pelleted down at 8000 RPM for 10 min. The pellet was resuspended in lysis-tris buffer (50 mM Tris, 10 mM EDTA, 150 mM NaCl, pH 7.5) that contained the protease inhibitor cocktail (Roche); two cycle freeze and thaw followed by the sonication. The sonicated sample was boiled in the water bath for 30 min. Next, samples were centrifuged at 16,000*g* for 30 min, and the supernatants were collected. Ten percent streptomycin sulfate (136 μl/ml) and glacial acetic acid (228 μl/ml) were added to the supernatant, followed by centrifugation at 16,000*g*, 10 min at 4 °C. The resulting supernatant was precipitated by an equal volume of saturated ammonium sulfate at 4 °C. Precipitated samples were washed with (NH4)2SO4 solution at 4 °C (saturated ammonium sulfate and water, 1:1 v/v). The washed pellet was then resuspended using 100 mM NH4(CH3COO) under constant stirring for 10 min. The protein was precipitated by the addition of an equal volume of absolute ethanol. Ethanol precipitation was repeated twice at room temperature. The collected protein was resuspended in 100 mM NH4(CH3COO), lyophilized, and stored at −20 °C for further chromatographic purification.

### Size-exclusion chromatography

Synuclein as well as A30P and A53T protein was dissolved in PBS buffer, pH 7.4 and centrifuged for 30 min at 14, 000*g* using a benchtop microcentrifuge (Eppendorf centrifuge 5424). Next, 500 μl of synuclein A30P and A53T concentrated protein was loaded on a Superdex 200 10/300 gel filtration column in AKTA pure (GE Healthcare) FPLC. Proteins were eluted isocratically with a flow rate of 0.5 ml/min at 4 °C using the same buffer 1.5 ml fractions were collected according to the UV-VIS detection at 280 nm.

### Liposome preparation

LUVs of DMPS, DOPS, POPS, and DSPS were prepared accordingly to the method proposed by Galvagnion *et al.* (47) First, lipids were dissolved inPBS, pH 7.4. Second, samples were heated in a water bath to ∼65 °C for 30 min. After that, samples were immersed in liquid nitrogen for 1 min. The procedure was repeated 8 to 10 times. Third, samples were passed through the extruder equipped with a 100 nm membrane (Avanti, Alabaster). The size of LUVs was determined using dynamic light scattering (Wyatt DynaPro NanoStar). All LUVs were ∼100 nm in diameter, Fig. S5. Zeta potentials of LUVs were determined using Malvern Zetasizer Nano ZS. Zeta potential of the LUVs ranged from −24.7 to −34.5 mV, Fig. S6.

### α-Syn, A30P, and A53T mutants aggregation

In the lipid-free environment, 100 μM of α-syn, A30P, and A53T mutants was dissolved in PBS; the solution pH was adjusted to pH 7.4. For DMPS, DOPS, POPS, and DSPS, 100 μM of α-syn, A30P, and A53T was mixed with an equivalent concentration of the corresponding LUVs, Fig. S7; the pH of the final solution was adjusted to pH 7.4 using concentrated HCl. Next, samples were placed into 96-well plate that was kept in the plate reader (Tecan, Männedorf) at 37 °C for 160 h under 510 rpm agitation. The excitation was set to 450 nm, and the emission was set to 490 nm for ThT kinetic.

### Kinetic measurements

Rates of protein aggregation were measured using ThT fluorescence assay. For this, samples were mixed with 2 mM of ThT solution and placed into 96-well plate that was kept in the plate reader (Tecan, Männedorf) at 37 °C for 160 h under 510 rpm agitation. Fluorescence measurements were taken every 10 min (excitation 450 nm; emission 488 nm). Each kinetic curve is the average of four independent measurements, Fig. S2.

### AFM imaging

Microscopic analysis of protein aggregates were performed on a AIST-NT-HORIBA system using silicon AFM probes (force constant 2.7 N/m; resonance frequency 50–80 kHz) purchased from AppNano. Preprocessing of the collected AFM images was made using AIST-NT software. Protein and protein-lipid solutions, suspended in a PBS buffer, were diluted in a 1:15 ratio of deionized water. Three 10 × 10 μm areas were analyzed per sample with 6 to 7 heights recorded from each before capturing the final image.

### Circular dichroism

After 140 h of protein incubation at 37 °C, samples were diluted using PBS and placed into quartz cuvette. CD spectra were measured immediately using Jasco J1000 CD spectrometer (Jasco). In total, three spectra were collected from each sample from 190 to 240 nm and then averaged.

### ATR-FTIR spectroscopy

After 140 h of protein aggregation at 37 °C, protein samples were deposited onto the crystal of 100 FTIR spectrometer (PerkinElmer) equipped with the ATR module. Samples were dried at room temperature; three spectra were collected from each sample and averaged.

### Atomic force microscopy-Infrared

AFM-IR spectra were collected on a Nano-IR3 system (Bruker, Santa Barbara) equipped with a QCL laser using gold-coated contact-mode AFM scanning probes (ContGB-G AFM probe, NanoAndMore). Collected spectra were averaged by three times and smoothed by Savitzky-Golay filter (second order) in MATLAB. Spectral deconvolution of averaged spectra was conducted in GRAMS/AI (https://www.thermofisher.com/order/catalog/product/INF-15000). The following wavenumbers were considered for the fitting of the secondary structure: parallel β-sheet at 1624 cm^−1^, α-helix, and random coil at 1655 cm^−1^, β-turn at 1682 cm^−1^, and antiparallel β-sheet at 1698 cm^−1^.

### Cell toxicity assays

Cell toxicity assays were conducted using the N27 rat dopaminergic neuron cell line. The cells were cultured in 96-well plates with RPMI 1640 medium supplemented with 10% fetal bovine serum (FBS) at 37 °C and 5% CO_2_. After reaching approximately 70% confluency following 24 h of incubation, the cells were ready for further experimentation.

To perform the LDH assay, 100 μl of the medium was replaced with an RPMI 1640 medium containing 5% FBS and 10 μl of the protein samples. The FBS concentration was reduced to decrease the baseline absorbance level of the analyzed samples. Following another 24 h of incubation, the CytoTox 96 cytotoxicity assay kit (G1781, Promega) was used to quantify the amount of LDH released into the cell culture medium. LDH is an enzyme present in the cytosol, and its release into the surrounding medium indicates damage to the plasma membrane.

The concentration of LDH was determined by measuring the conversion of lactate to pyruvate through NAD+ reduction to NADH. This reduction then facilitated the conversion of a tetrazolium salt to a red formazan product with an absorption maximum of 490 nm. The level of formazan produced directly correlated with the amount of LDH released, providing a measure of the toxicity of the protein aggregates to the N27 cells.

## Data availability

Data will be available upon the reasonable request from the authors.

## Supporting information

This article contains supporting information.

## Conflict of interest

The authors declare that they have no conflicts of interest with the contents of this article.
