# Supplementary material for: The toxicities of A30P and A53T α-synuclein fibrils can be uniquely altered by the length and saturation of fatty acids in phosphatidylserine
Source: J Biol Chem. 2023 Oct 26;299(12):105383. doi: 10.1016/j.jbc.2023.105383 (PMC10679493; doi:10.1016/j.jbc.2023.105383)
Supplement: Supporting Figures S1–S7 [file mmc1.docx]

The Toxicities of A30P and A53T α‑Synuclein Fibrils Can be Uniquely Altered by The Length and Saturation of Fatty Acids in Phosphatidylserine

Abid Ali^1^, Kiryl Zhaliazka,^1^ Tianyi Dou,^1^ Aidan P. Holman,^1,2^ and Dmitry Kurouski^*1,3^

1. Department of Biochemistry and Biophysics, Texas A&M University, College Station, Texas 77843, United States

2. Department of Entomology, Texas A&M University, College Station, Texas 77843, United States

3. Department of Biomedical Engineering, Texas A&M University, College Station, Texas, 77843, United States

Supporting Information

Figure S1. Averaged ThT aggregation kinetics of WT, A30P and A53T aggregation in the lipid-free environment (blue, WT; light blue, A30P; and purple, A53T) and in the presence of POPS (pink), DOPS (red), DMPS (yellow) and DPSP (green) at 1:1 molar ratio.

Figure S2. Individual runs of ThT aggregation kinetics of WT, A30P and A53T aggregation in the lipid-free environment (blue, WT; light blue, A30P; and purple, A53T) and in the presence of POPS (pink), DOPS (red), DMPS (yellow) and DPSP (green) at 1:1 molar ratio. For each experiment, four individual samples (red, blue, green and purple) were analyzed.

Figure S3. Individual (grey) and averaged (black) AFM-IR spectra acquired from WT, A30P and A53T fibrils grown in the lipid-free environment, as well as in the presence of DOPS, POPS, DMPS and DSPS.


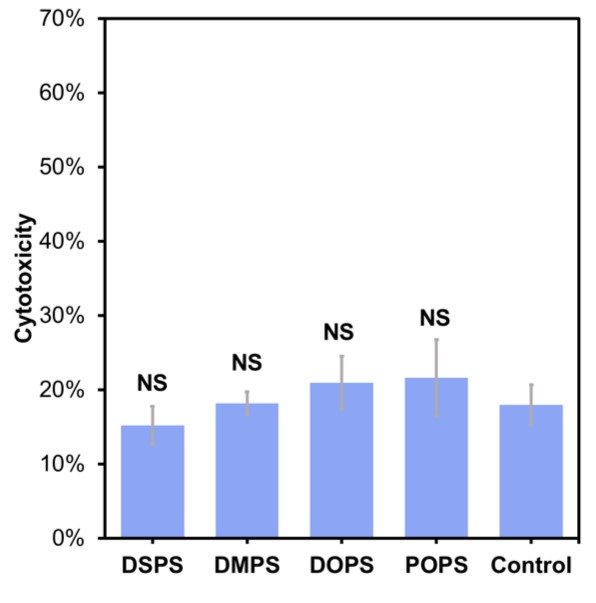


Figure S4. Histograms of LDH assays of DSPS, DMPS, DOPS, and POPS LUVs.

Figure S5. A histogram of LUV diameters according to the DLS measurements.

Figure S6. A histogram of zeta potentials of LUVs of DMPS, DOPS, DSPS, and POPS.

Figure S7. AFM images of LUVs of DMPS, DOPS, DSPS, and POPS. Scale bars are 500 nm.
